# Genomic analysis of the 2017 Aotearoa New Zealand outbreak of *Mycoplasma bovis* and its position within the global population structure

**DOI:** 10.3389/fmicb.2025.1600146

**Published:** 2025-07-23

**Authors:** Barbara M. Binney, Edna Gias, Jonathan Foxwell, Alvey Little, Patrick J. Biggs, Nigel French, Callum Lambert, Hye Jeong Ha, Glen P. Carter, Miklós Gyuranecz, Bart Pardon, Sarne De Vliegher, Filip Boyen, Jade Bokma, Volker Krömker, Nicole Wente, Timothy J. Mahony, Justine S. Gibson, Tamsin S. Barnes, Nadeeka Wawegama, Alistair R. Legione, Martin Heller, Christiane Schnee, Sinikka Pelkonen, Tiina Autio, Hidetoshi Higuchi, Satoshi Gondaira, Michelle McCulley

**Affiliations:** ^1^Genomics Team, Animal Health Laboratory, Biosecurity New Zealand, Upper Hutt, New Zealand; ^2^Bacteriology & Mycology, Animal Health Laboratory, Biosecurity New Zealand, Upper Hutt, New Zealand; ^3^Virology Mammalian, Animal Health Laboratory, Biosecurity New Zealand, Upper Hutt, New Zealand; ^4^Molecular Epidemiology & Public Health Laboratory (mEpiLab), Tāwharau Ora, School of Veterinary Science, Massey University, Palmerston North, New Zealand; ^5^School of Food Technology and Natural Sciences, Massey University, Palmerston North, New Zealand; ^6^Tāwharau Ora, School of Veterinary Science, Massey University, Palmerston North, New Zealand; ^7^Bioprocessing and Fermentation, Biotechnologies, Callaghan Innovation, Lower Hutt, New Zealand; ^8^Department of Microbiology and Immunology, Peter Doherty Institute, University of Melbourne, Melbourne, VIC, Australia; ^9^HUN-REN Veterinary Medical Research Institute, Budapest, Hungary; ^10^Department of Internal Medicine, Reproduction, and Population Medicine, Faculty of Veterinary Medicine, Ghent University, Merelbeke, Belgium; ^11^M-team & Mastitis and Milk Quality Research Unit, Department of Internal Medicine, Reproduction, and Population Medicine, Faculty of Veterinary Medicine, Ghent University, Merelbeke, Belgium; ^12^Department of Pathobiology, Pharmacology and Zoological Medicine, Faculty of Veterinary Medicine, Ghent University, Merelbeke, Belgium; ^13^Department of Internal Medicine, Reproduction, and Population Medicine, Faculty of Veterinary Medicine, Ghent University, Merelbeke, Belgium; ^14^Department of Veterinary and Animal Sciences, Section for Production, Nutrition and Health, Faculty of Health and Medical Sciences, University of Copenhagen, Frederiksberg, Denmark; ^15^Department of Bioprocess Engineering and Microbiology, Faculty II, Hannover University of Applied Sciences and Arts, Hanover, Germany; ^16^The University of Queensland, Queensland Alliance for Agriculture and Food Innovation, St Lucia, QLD, Australia; ^17^School of Veterinary Science, The University of Queensland, Gatton, QLD, Australia; ^18^Asia-Pacific Centre for Animal Health, Melbourne Veterinary School, Faculty of Science, The University of Melbourne, Melbourne, VIC, Australia; ^19^Institute of Molecular Pathogenesis, Friedrich-Loeffler-Institut, Federal Research Institute for Animal Health, Jena, Germany; ^20^Animal Health Diagnostic Unit, Laboratory and Research Division, Finnish Food Authority, Kuopio, Finland; ^21^Animal Health Laboratory, Department of Health and Environmental Sciences, School of Veterinary Medicine, Rakuno Gakuen University, Ebetsu, Japan

**Keywords:** *Mycoplasma bovis*, multilocus sequence typing (MLST), whole genome MLST, core genome MLST, outbreak, genomic epidemiology

## Abstract

In 2017 an outbreak of *Mycoplasma bovis* (*M. bovis*), an infectious agent of cattle, was identified in Aotearoa New Zealand. This study characterizes the genomic population structure of the outbreak in New Zealand and compares it with the known global population structure using multilocus sequence typing (MLST) and genomic analysis. The New Zealand outbreak strain was MLST genotyped as ST21. A comprehensive collection of 840 genomes from the New Zealand outbreak showed a pattern of clonal expansion when characterized by MLST, core genome MLST (cgMLST) and whole genome MLST (wgMLST). A lineage of genomes was found with no *in silico* identifiable *pta2* locus, a housekeeping gene used in the MLST scheme. We compared a sample set of 40 New Zealand genomes to 47 genomes from other countries. This group had 79 ST21 genomes and eight genomes that were single nucleotide polymorphism (SNP) variants within the MLST loci of ST21. Two of the 47 international genomes showed signs of extensive unique recombination. Unique alleles in six genes were identified as present only in the New Zealand genomes. These novel variants were in the genes; haeIIIM encoding for cytosine-specific methyltransferase, *cys*C encoding for cysteinyl tRNA synthetase, era encoding for GTPase Era, *met*K encoding for S-adenosylmethionine synthase, *par*E encoding for DNA topoisomerase, and *his*S encoding for histidine-tRNA ligase. This finding could be due to a population bottleneck, genetic drift, or positive selection. The same sample set of 40 New Zealand genomes were compared using MLST to 404 genomes from 15 other countries and 11 genomes without a known country. A FastBAPS analysis of 455 genomes showed a global population structure with 11 clusters. Some countries, such as Canada, Denmark and Australia contained both internally closely related genomes and some genomes that were more closely related to genomes found in other countries. Our results support the need for Whole Genome Sequencing (WGS) as well as MLST genotyping in *M. bovis* outbreaks. They also support the importance of understanding the national and international movement patterns of cattle and their genetic material, as possible routes of transmission, when managing the spread of *M. bovis*.

## 1 Introduction

*Mycoplasma bovis* (*M. bovis*) is an important pathogen of cattle. While mainly associated with respiratory disease, it can also cause mastitis, arthritis, and otitis media (Perez-Casal, 2020). Resistance to current antibiotic therapy is increasing in *M. bovis* and without an effective vaccine any plan to treat and control is difficult to implement (Gautier-Bouchardon et al., 2014). *M. bovis* is widespread and considered a significant burden on the cattle industry in Europe and North America, causing economic losses due to reduced milk production, treatment costs, and animal deaths (Maunsell et al., 2011; Calcutt et al., 2018).

*M. bovis* was first discovered in the USA as a cause of bovine mastitis in 1961 (Hale et al., 1962). Since then, *M. bovis* appears to have spread around the world, reaching Australia in 1970, Europe in the mid-1970s, South America and Japan in the 1980s, and Ireland in 1994 (Doherty et al., 1994; Nicholas and Ayling, 2003; Dudek et al., 2020). With the advent of more sensitive molecular diagnostic tools in the following decades, these fastidious mycoplasmas were also detected in China, India and Africa (Dudek et al., 2020). Currently, *M. bovis* is considered to appear world-wide with differing prevalences. In New Zealand, *M. bovis* was first reported in July 2017 on a farm in the South Island (Jordan et al., 2021), prior to this it had been considered free of the disease (McDonald et al., 2009).

New Zealand is geographically isolated and most of the species used in modern agriculture were only introduced in the last 250 years (Binney et al., 2014). After *M. bovis* was detected in New Zealand in 2017, an eradication program was implemented in 2018 (Jordan et al., 2021). This program involved extensive testing to detect *M. bovis* and included depopulation of cattle from infected properties.

Despite its small genome (∼1 Mbp), *M. bovis* shows significant variation particularly in some regions, such as variable membrane surface lipoprotein (*vsp*) genes, integrative conjugative elements (ICE) and insertion sequences (IS) (Aebi et al., 2012; Qi et al., 2012; Tardy et al., 2015). Recombination events, including Mycoplasma chromosomal transfer (MCT), contribute to the diversity which is reflected by having an open pangenome (Dordet-Frisoni et al., 2014; Garcia-Galan et al., 2022). Some genes thought essential in other *Mycoplasma* species, e.g., DnaJ, have been shown to be “dispensable genes” in the *M. bovis* genome (Sharma et al., 2014). There is evidence of strain variation in new hosts, such as North American bison (*Bison bison*) and American pronghorn (*Antilocapra americana*) (Register et al., 2018; Malmberg et al., 2020).

The global population structure of *M. bovis* is influenced by numerous factors (Kumar et al., 2020; Tardy et al., 2020; Yair et al., 2020). The potential routes of animal-to-animal transmission are: colostrum, milk, semen, airborne droplets, and intrauterine transmission (Dudek et al., 2020), with the potential inclusion of transmission by fomites (Piccinini et al., 2015). On farm practices like animal husbandry (Pfützner and Sachse, 1996; Spergser et al., 2013) and the widespread use of antibiotics (Becker et al., 2015; Tardy et al., 2020) can also have an effect. The international movement of live cattle and breeding material has a role (Haapala et al., 2018; Dudek et al., 2020; Yair et al., 2020). Transmission by infected semen has been reported (Haapala et al., 2018), but recent work suggests transmission from infected embryos presents a low-level risk (Pohjanvirta et al., 2023).

Previous genomic studies support cattle movement, and management practices as affecting the population structure of *M. bovis*. An investigation into the effect of the international cattle trade on *M. bovis* entering Israel found genomes from Europe formed a separate cluster to those from Australia and China, while genomes from Israel and the USA were found in both clusters (Yair et al., 2020). Yair et al. (2020) considered the clustering of China and Australia was a result of the export of live cattle from Australia to China. Kumar et al. (2020) suggested the shared genomic diversity between Canada and the USA isolates was due to shared cattle movements (Kumar et al., 2020). Most of the currently published Australian genomes are from a single widely distributed strain (Parker et al., 2016). On a global scale, the correlation between country and genotype is poor (Garcia-Galan et al., 2022). Genomic analysis in some Nordic countries and genotyping in France, suggest emerging dominant strains driven by antibiotic resistance (Becker et al., 2015; Tardy et al., 2020). In Austria, a strain caused an outbreak in 2007 and then re-emerged in 2009; this was related to the practice of shared grazing by different species (Spergser et al., 2013).

Since its inception in 1998, the MLST genotyping of bacteria has been widely used in strain identification and reconstruction of clonal relationships (Maiden et al., 2013; Jolley et al., 2018). The approach indexes a section of sequence from a housekeeping gene (a locus). A set of housekeeping genes produce multiple loci. The combined loci indexes create unique profiles called sequence types (ST). The housekeeping genes are used as they are considered essential genes that are related to basic cellular functions. They are highly conserved, giving the MLST approach stability as a typing system, but still provide enough sequence variation to be informative. The exact number of housekeeping genes (loci) used for a bacterial species MLST scheme can vary, but it is often seven loci. In our analysis we used the seven loci MLST scheme developed and updated by Register et al. (2015); Register et al. (2020) for *M. bovis* and available on PubMLST,^1^ although other systems exist (Rosales et al., 2015; Bell-Rogers et al., 2018; Jolley et al., 2018). This approach has also been expanded to include MLST of the core genome (cgMLST) (Menghwar et al., 2022) and MLST of the core genome and accessory genes (whole genome MLST/wgMLST) (Uelze et al., 2020).

This study provides a comprehensive analysis of the genomic population structure of *M. bovis*, both global strains and those within the 2017 New Zealand outbreak. We identify and discuss the genomic variations in New Zealand *M. bovis* compared to genomes from other countries of the same MLST (or one SNP variants). Finally, using a substantial genomic dataset from the 2017 outbreak we show how an outbreak of *M. bovis* behaves in a naive population when national eradication, by testing and herd culling, is undertaken rather than attempting to control it with animal husbandry techniques and antibiotics.

## 2 Methods and materials

### 2.1 Sample collection

#### 2.1.1 Sample collection in New Zealand

Samples were collected from infected cattle using swabs from a range of anatomical sites including nasal and oropharyngeal cavities, pharyngeal tonsils, as well as samples from synovial fluid, lung tissue and milk.

We analyzed the genomes (*n* = 840) from the New Zealand *Mycoplasma bovis* ST21 outbreak. Samples were collected between 19 July 2017 and 17 February 2022 from 14 of the 27 regions around New Zealand viz. Canterbury, Nelson, North Canterbury, Northland, Otago Lakes, South Canterbury, Southland, Taranaki, Taupo, Waikato, Wairarapa, Whanganui, Wellington, and Westland. By January 2022, there had been a total of 272 confirmed infected properties. We have one genome from each of 111 infected properties, with some properties contributing multiple genomes.

#### 2.1.2 Collection and processing of samples from outside of New Zealand

444 genomes were obtained from outside of New Zealand. Of these, 163 genomes were from samples or sequences collected in 10 countries and 281 sequences were downloaded from NCBI’s sequence read archive (SRA).^2^ The 163 genomes came from a range of sampled anatomical sites included upper and lower respiratory sites, as well as synovial fluids, and milk from infected cattle. The methods used to culture and sequence these isolates are summarized in Supplementary Table 3 (Sulyok et al., 2014; Haapala et al., 2018; Zhu et al., 2018; Tardy et al., 2020; Triebel et al., 2023). Information on all 444 sequences and their availability from NCBI is in Supplementary Table 8.

### 2.2 Culture of New Zealand isolates

Samples were inoculated in Friis broth (FB) and processed through a series of serial dilutions, filtrations (0.45 μm) and inoculations onto Friis agar (FA), at 3–4 days intervals to isolate single colonies. Samples were incubated at 37°C, 5% CO_2_. A single isolated colony of *M. bovis* was chosen and inoculated in FB, then the DNA was extracted after 3–4 days incubation. Jaramillo et al., 2023 describe the preparation methods of the FB and FA (Jaramillo et al., 2023).

### 2.3 Whole genome sequencing of New Zealand *Mycoplasma bovis* isolates

For New Zealand isolates of *M. bovis*, DNA was extracted from 2 to 3 mL of the culture using the QIAmp DNA Mini Kit (QIAGEN, Hillden, Germany) according to the manufacturer’s instruction (extraction from tissue protocols).

For all isolates extracted DNA concentration was measured by Qubit dsDNA High Sensitivity (HS) Assay kit (Thermo Fisher Scientific, Eugene, Oregon, United States) and the quality was assessed by NanoDrop spectrophotometer (Thermo Fisher Scientific, Wilmington, Delaware, United States). qPCR was performed on the samples using the VetMax™ *M. bovis* kit (Life Technologies, Carlsbad, CA, United States) to confirm the presence of *M. bovis* DNA. DNA libraries were prepared using 1 ng of *M. bovis* DNA with the Nextera XT DNA Library Preparation Kit (Illumina, San Diego, CA, United States) following the manufacturer’s protocol. The concentrations and quality of the purified libraries were assessed using the Qubit dsDNA HS Assay kit and HS DNA kit on an Agilent 2100 Bioanalyzer or the HS D5000 ScreenTape kit on the 4200 TapeStation (Agilent Technologies, Waldbronn, Germany). Each DNA library was normalized and then pooled to an equimolar concentration of 2 nM. Following denaturation using 0.2 N NaOH, the pooled libraries were diluted further to 10 pm and spiked with 15% PhiX Sequencing Control V3 (Illumina) following Illumina’s recommendation for low diversity libraries. Sequencing was performed on the spiked libraries on an Illumina MiSeq using the MiSeq reagent Kit v2 (500 cycles) (Illumina).

### 2.4 Bioinformatic analysis

**FIGURE 1:**
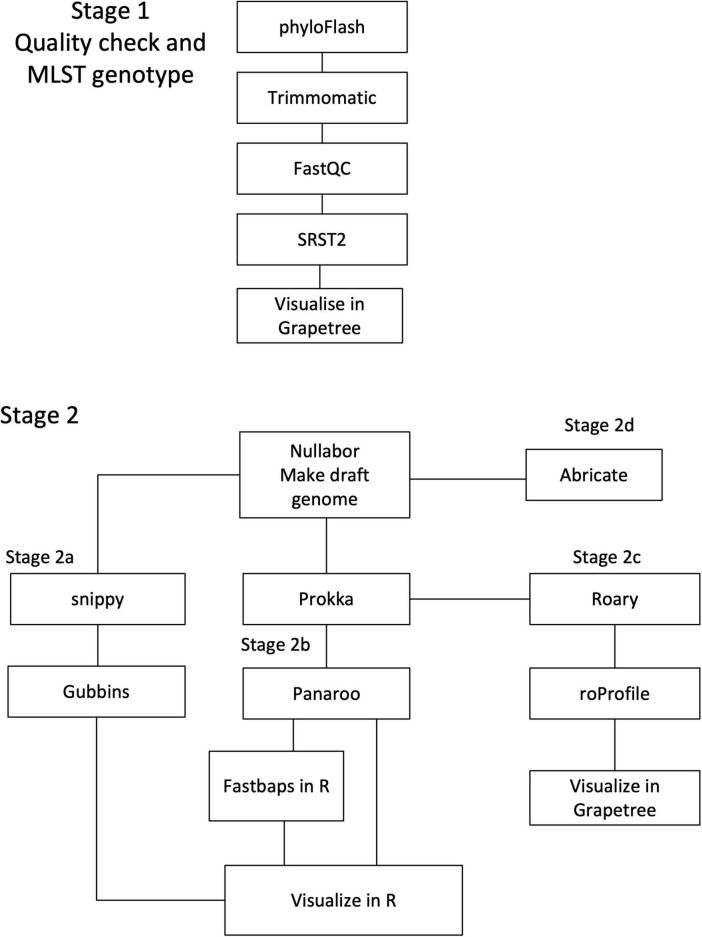
Workflow of Bioinformatic analysis. The workflows followed in analyzing the three genomic datasets. All samples followed Stage 1 for sequencing quality checks and MLST genotyping. In the second Stage, the 2a workflow is for SNP variance and recombination analysis. The 2b workflows includes core gene alignment analysis with the option of using FastBAPS for population structure. Stage 2c produces the core genome MLST and whole genome MLST. Stage 2d uses Abricate to check the genomes for putative adhesion- and virulence-related genes.

The genomic data was divided into 3 datasets (see 4.3 for details), which were each analyzed separately. The workflows used in this paper are outline in Figure 1.

#### 2.4.1 Initial sequencing quality check and *in silico* genotyping

First, the raw sequences were checked for bacterial contamination by phyloFLASH v3.31b1 (Gruber-Vodicka et al., 2020). Trimmomatic v0.39 trimmed nucleotides at both ends with a Phred score < 15 and removed any detected Illumina adapters (Bolger et al., 2014). A sliding window approach was used to find consecutive 4 bp segments with an average quality score < 15, at which point the reads were trimmed. Reads shorter than 100 bp after trimming were removed by Trimmomatic v0.39 (Bolger et al., 2014). The resulting files were evaluated by FastQC v0.11.9 (Andrews, 2010) and reviewed using MultiQC v1.8 (Ewels et al., 2016). Multilocus Sequence Typing (MLST) was undertaken using SRST2 v0.2.0 (Inouye et al., 2014). The pubMLST (Jolley et al., 2018) profiles were downloaded on 21 July 2022 for this study (Register et al., 2020). New alleles and profiles were submitted to pubMLST for naming. The results of the MLST analysis were visualized in grapetree (Zhou et al., 2018). All the genomic data was assessed for quality and genotyped as summarized in Figure 1 as Stage 1.

#### 2.4.2 Genome assembly

The Nullarbor v2.0.20191013 pipeline assembled all the sequences into draft genomes (Seemann et al., 2019). The Nullarbor pipeline performs an array of analyses, but only the draft genome assemblies produced by SKESA v2.4.0 were used (Souvorov et al., 2018). The genome assembly is summarized as Stage 2 in Figure 1.

#### 2.4.3 Genome datasets

The draft genomes were grouped into three genomic datasets, and each were analyzed separately to compare distinct characteristics of the outbreak.

The first dataset initially contained international genomes (*n* = 484) from 16 countries including 11 from unknown countries and a sample set of 40 genomes from New Zealand. The dataset was checked for quality then MLST genotyped (Figure 1, Stage 1), investigated for population structure (Figure 1, Stages 2b) and for virulence genes (Figure 1, Stage 2d).

The second dataset (*n* = 87) was a subset of the international dataset containing only ST21 and one SNP variants of this ST (Supplementary Table 4). There are 40 New Zealand genomes, 47 from 9 countries, and one without a known source country. The genes in the core genome were also compared to identify allelic variants specific in the New Zealand genomes and examined for recombination events (Figure 1, Stages 2a, 2b without FastBAPS and 2c).

The third dataset was the New Zealand genomes (*n* = 840) (Supplementary Table 2), which were characterized using MLST, cgMLST and wgMLST analysis (Figure 1, Stage 1, 2b Panaroo only, and 2c).

#### 2.4.4 Core-genome MLST and whole genome MLST

The assembled draft genomes were annotated in Prokka v1.14.6 (Seemann, 2014) using Translation table 4, then a pangenome was generated in Roary v3.13.0 (Tange, 2011; Page et al., 2015). The Roary pangenome was checked for quality and some genes were discarded by roProfile v1.4.5 (Mendes, 2016). Then roProfile made a core genome multilocus sequence type (cgMLST) and a whole genome multilocus sequence type (wgMLST). The cgMLST and wgMLST were visualized in grapetree v2.2 as minimum spanning trees (MST) using the MSTreeV2 algorithm (Zhou et al., 2018). The preceding steps are represented in Stage 2c in Figure 1. Using the cgMLST profiles, six allelic variants in the New Zealand genomes were identified, the sequence alignments were checked in Jalview v2.11.1.4 (Waterhouse et al., 2009) and the genes confirmed by UniProt (Zaru et al., 2023).

#### 2.4.5 Core gene alignment analysis

The Prokka annotated genomes were used in Panaroo v1.2.10 (Tonkin-Hill et al., 2020) to make an alignment of the core genes using default settings. Panaroo identified a set of core genes defined as those genes present in 99-100% of the genomes in the dataset. Panaroo also made a pangenome from the dataset. A distance matrix based on the number of sites with a base pair difference (SNP) in the core gene alignment was estimated and made into a neighbor joining tree using R v4.1.2 package ape v5.7-1 (Paradis et al., 2004; R Core Team, 2023). The results of the analysis of the core genes were also used to identify the sequence variant alleles in the New Zealand *M. bovis* genomes. This workflow is included in Stage 2b in Figure 1.

#### 2.4.6 Population structure

FastBAPS v1.0.8 (Tonkin-Hill et al., 2019) was used for clustering the core gene alignment for the dataset of international genomes. FastBAPS identified an approximate fit to a Dirichlet process mixture model for clustering using the optimized symmetric prior. This is an option in Stage 2b in Figure 1 of the workflow.

#### 2.4.7 SNP and recombination analysis

The ST21 dataset (87 genomes) was examined to estimate genomic recombination and SNP variants. Snippy v4.4.3^3^ is a pipeline that used the Burrows-Wheelers Aligner v0.7.17-r1188 (Li, 2013) and SAMtools v1.9 (Danecek et al., 2021) to align reads from draft genomes to the New Zealand reference genome; NZ_B0132. Snippy also includes FreeBayes v1.3.2 (Garrison and Marth, 2012) to identify variants among the alignments. Gubbins v2.3.4 estimated the number and position of recombination events and used RAxML to make a maximum likelihood tree (Stamatakis, 2014; Croucher et al., 2015). In R v4.1.2 using a script with RCandy v1.0.0 (Chaguza et al., 2022; R Core Team, 2023) the positions of the recombination events estimated by Gubbins were represented along each genome (Figure 1, Stage 2a workflow).

#### 2.4.8 Detection of putative adhesion- and virulence-related genes

A bespoke database was made for Abricate v1.0.1 (https://github.com/tseemann/abricate) using the 91 putative adhesion- and virulence-related genes identified in *M. bovis* by Josi et al. (2019). The genes present in the ST21 dataset (*n* = 87) were compared to those found in the rest of the international dataset. The presence or absence of the sequences from Josi et al. (2019) in each genome is listed in Supplementary Table 7 (Figure 1, Stage 2d workflow).

#### 2.4.9 Generation of a complete reference genome

A reference genome was chosen by sequencing a representative New Zealand isolate NZ_B0132 (NCBI CP192245.1). *M. bovis* was pelleted from 1 L of 3–4 days old culture in FB by centrifugation at 3,000 × g for 30 min at 4°C. Genomic DNA was extracted using the method described above for the other New Zealand isolates but with the addition of 28 U RNase A (Qiagen) at room temperature for 5 min during the lysis stage. To meet the sample requirements for PacBio long-read sequencing, an additional isopropanol precipitation step was performed to concentrate the eluted genomic DNA. Sodium acetate was added to a final concentration of 0.3 M (pH 5.2), followed by 0.7 volumes of room-temperature isopropanol. The mixture was centrifuged immediately at 15,000 × g for 30 min at 4°C. The supernatant was carefully removed, and the DNA pellet was washed with room-temperature 70% ethanol to remove residual salts and facilitate resuspension. A second centrifugation was performed at 15,000 × g for 15 min at 4°C, after which the ethanol was decanted and the pellet air-dried for 20 min. The DNA was then resuspended in 10 mM Tris-HCl, pH8.0 (Fisher Scientific, Fair Lawn, New Jersey, United States).

Sample quality was assessed as described above, with the addition of genomic integrity analysis using pulsed-field gel electrophoresis (PFGE). High molecular weight genomic DNA was confirmed using the Bio-Rad CHEF Mapper XA system, following the PacBio protocol “Using the BIO-RAD CHEF Mapper XA Pulsed Field Electrophoresis System”.^4^

The purified DNA was sequenced on a Pacific Biosciences, Inc., RS II platform using P6-C4 chemistry according to the 20 kb Template Preparation and the BluePippin DNA Size Selection system protocol (Pacific Biosciences, Inc.). Assembly of the complete genome of NZ_B0132 was performed using SMRT analysis system v2.3.0.140936 (Pacific Biosciences). Raw sequence data were *de novo* assembled using the HGAP3 protocol with a minimum seed read length of 1 kb, a minimum read quality of 0.80, predicted genome size of 5 Mb, target coverage of 10 and over-lapper error rate of 0.04. Polished contigs were further error corrected using Quiver v1. The read alignments were visually assessed and ordered contigs joined in Geneious v8.1.5.^5^ The final assembly structure was checked by mapping raw reads against the alignment with BridgeMapper v1 in the SMRT analysis system.

## 3 Results

### 3.1 Global population structure of *Mycoplasma bovis*

The genomes of *M. bovis* isolates (*n* = 484) were collected from 16 different countries and *n* = 11 *M. bovis* genomes without known countries of origin. They were assembled using the Nullarbor 2 pipeline. Within this dataset was a subset of 40 genomes from the New Zealand outbreak. Note that 29 draft genomes were excluded from additional analysis because when assembled they had 300 or more contigs, or they did not contain between 680 and 850 genes when annotated. A core gene alignment of 455 *M. bovis* isolates revealed a core genome length of 695,994 bp containing 563 genes.

**FIGURE 2:**
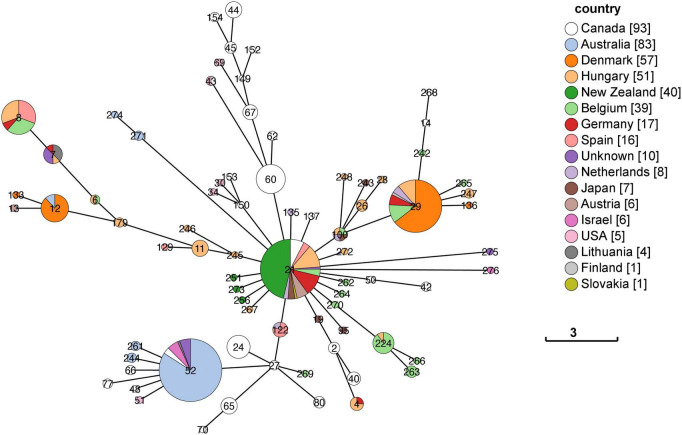
Minimum spanning tree of the MLST distribution of *M. bovis* by country. The MST of the 7-gene MLST scheme developed by Register et al. (2020) showed the ST distribution for 444 genomes across 16 countries and 10 from an unknown country. Each of the STs are represented as a node, and each node is colored in proportion to the number present from each country. The legend has a tally in square brackets of the total genomes from each country. Several countries (including Australia, Denmark, and Canada) have STs that are quite different to each other, e.g., Denmark has ST29 and ST12 genomes. The branch length between nodes represents the number of loci that differ between each ST node. The scale bar shows the distance for differences at three of the 7-loci.

Initially, the sequences for n = 484 isolates from the international dataset were genotyped for their MLST sequence type (ST) but *n* = 40 isolates were removed from the final analysis as they either lacked an identifiable locus (*n* = 11) or their draft genome was not of sufficient quality (*n* = 29). A full profile with all 7 loci is needed for a complete sequence type identity. The distribution of the remaining 444 STs by country is shown as a minimum spanning tree (Figure 2).

**FIGURE 3:**
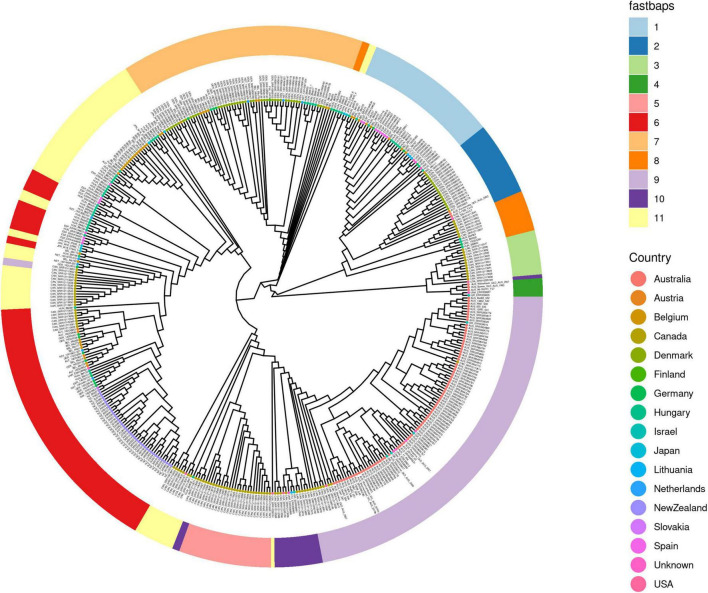
The population structure of 455 *M. bovis* genomes using FastBAPS clustering. A FastBAPS clustering analysis was made using the international dataset core gene alignments for 455 genomes from 16 countries and 11 from an unknown country of origin (Supplementary Table 1). The tips of the neighbor-joining tree are color-coded according to the country in which the genomes were found. The branch lengths are all equivalent. The FastBAPS clusters are in the outer ring and divide the genomes into 11 clusters. The FastBAPS cluster for each genome is designated by a color in the ring and aligns to the genome at the tip of the rooted neighbor joining tree. Some of the FastBAPS clusters, e.g., 5 and 1 are in a single clade, while some, e.g., 10 and 11 are not. The countries with larger sample sizes tend to have genomes more widely spread around the tree, e.g., Australia and Canada. The neighbor-joining tree is based on a distance matrix derived from SNP differences in the core gene alignment.

The population structure shown by the FastBAPS analysis of the core gene alignment for 455 genomes divided them into 11 clusters spread around the neighbor-joining tree (Figure 3). Several countries with larger sample sizes were distributed around the tree and between FastBAPS clusters. We identified five genomes, separate from the main Australian grouping (*n* = 82) in cluster 9, which are mainly ST52. There were two ST12 genomes (AUS_3-1355_TV10, AUS_Purrawunda_QLD_AUS_2003) in cluster 2. Cluster 4 contained two ST271 (AUS_99-193731_TV7, AUS_Bowen_QLD_AUS_1993) and one ST274 (AUS_Willowbank_QLD_AUS_2001). All five genomes were separate from the previously described Australian outbreak of ST52 by MLST profile and WGS (Parker et al., 2016). The Danish genomes divided into two large FastBAPS clusters, cluster 7 (*n* = 41) with mainly ST29 genomes and cluster 2 (*n* = 16) containing mainly ST12 genomes. The Canadian genomes (*n* = 94) were spread across seven clusters (3, 5, 6, 8, 9, 10, 11). Consistent with their MLST profiles, all five Canadian ST67 genomes were in cluster 5 and separate from the ST24 genomes (*n* = 12) in cluster 11. Cluster 9 includes ST52 from Canada (*n* = 3), Israel (*n* = 52) and Australia (*n* = 74).

Overall, when the core gene alignment SNP differences were visualized as a neighbor-joining tree and the FastBAPS clustering analysis included (Figure 3 and Supplementary Table 1) the results were consistent with the pattern seen in using MLST profiles (Figure 2)—where countries with larger sample sizes contained both closely and distantly related genomes. This pattern of within country diversity also shows some genomes being more closely related to genomes in another country.

A comparison was made of the putative adhesion- and virulence-related genes found by Abricate in the international dataset (*n* = 455) to see if there was a difference between the ST21 dataset (*n* = 87) and the rest. Altogether, 89 of the 91 genes in the *M. bovis* Abricate database were found. The 2 genes that were missing from both groups were IS1634AV transposase proteins. Four genes were found only in the non-ST21 group, but they occurred very infrequently (>10×); three were IS1634AV transposase proteins and one was a variable surface protein G (*vsp*). One gene, a variable surface protein antigen, was only present in the ST21 group but it was only present in 1 genome. The genes as identified by Josi et al. (2019) are listed in Supplementary Table 7.

### 3.2 Comparison of ST21 and closely related genomes

There were 87 genomes included in this analysis: 40 from New Zealand, 47 from nine other countries and one with an unknown country of origin. This group included n = 79 ST21 genomes and eight genomes that varied from ST21 by only one SNP across all loci in the 7-gene MLST scheme (Supplementary Table 4). The isolates (STs) that are one SNP variants to ST21 are NZ_M0102 (ST251), NZ_0U036 (ST256), BEL_Mb31 (ST262), BEL_Mb177 (ST264), HUN_BM632 (ST267), BEL_Mb205 (ST270), HUN_BM621 (ST272), and NZ_E0029 (ST273).

The core gene alignment identified in Panaroo for the 87 genomes was 777,194 bp long and contained 577 core genes. The wgMLST generated in roProfile contained 1004 loci, and the Panaroo generated pangenome contained 921 genes.

**FIGURE 4:**
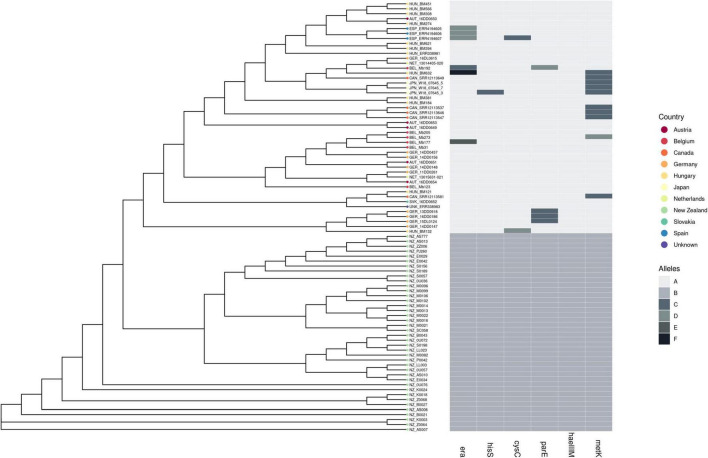
Comparison of 87 of *M. bovis* genomes (ST21 and one SNP variants) and six genes with allelic variants. A comparison of ST21 (*n* = 79) and one SNP variants of ST21 (*n* = 8) from a dataset composed of 40 NZ genomes and 47 other country genomes. The neighbor-joining tree was made from a distance matrix based on SNP differences between the core gene alignments. The branch lengths are equal. Six genes (*era*, *his*S, *cys*C, *par*E, *hae*IIIM, and *met*K) were identified as showing one or more sequence variants (alleles) present only in the New Zealand genomes and different to the sequences found in the ST21 overseas genomes.

Examination of the wgMLST and core gene alignment revealed that the New Zealand *M. bovis* genomes had unique allelic variants in six genes (Table 1). The different alleles identified in each genome are shown in Figure 4.

**TABLE 1:** The six genes in the ST21 (*n* = 87) dataset of core genes with unique sequences for New Zealand *M. bovis* genomes.

Gene	Protein name	Enzyme commission number
*hae*IIIM	Cytosine-specific methyltransferase	2.1.1.37
*cys*C	Cysteinyl tRNA synthetase	6.1.1.16
*Era*	GTPase Era	
*met*K	S-adenosylmethionine synthase	2.5.1.6
*par*E	DNA topoisomerase	5.6.2.2
*his*S	Histidine-tRNA ligase	6.1.1.21

**FIGURE 5:**
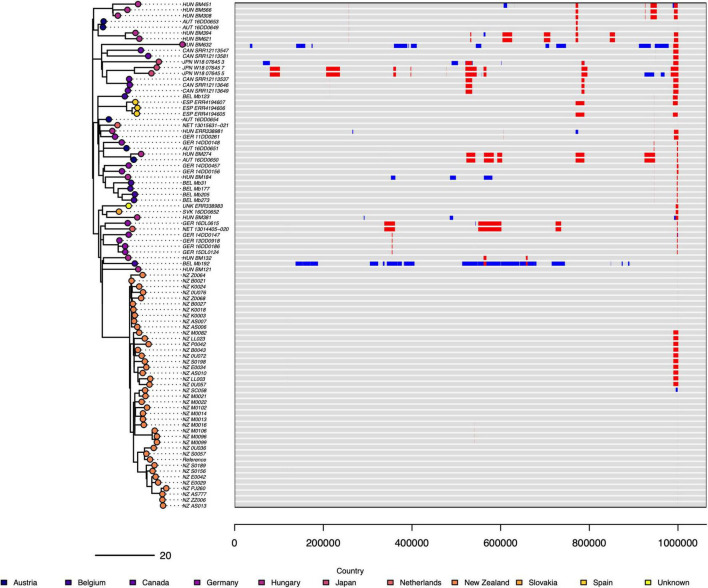
Estimated recombination events in of 87 *M. bovis* genomes (ST21 and one SNP variants). The putative recombination events detected by Gubbins in 87 genomes, is visualized by aligning the genomes against a reference genome on the right and comparing them in a phylogenetic tree on the left. The New Zealand reference genome (NZ_B0132) is 1,064,188 bp. The size and position of recombination events are in red and blue. Red indicates the recombination event was found in multiple genomes and blue signals the recombination event was limited to one genome that is present. There is variation in the position and size of estimated recombination events across the group of genomes. Two genomes (HUN_BM632, BEL_Mb192) show large areas of their genome are affected by these putative unique recombination events. The phylogenetic tree on the left is a maximum likelihood tree produced in Gubbins, and it shows the sample of 40 NZ genomes are closely related and have few identified recombination events.

Overall, at the genomic level the New Zealand *M. bovis* were genetically very similar to each other, and to a lesser extent similar to the other genomes in the 87-genome dataset. However, there is evidence of significant amounts of recombination (Figure 5) in the genomes of Bel_Mb192 (ST21) and Hun_BM632 (ST267, a one SNP MLST variant of ST21). The genes at the fully or partially affected loci when Bel_Mb192 and Hun_BM632 are aligned to NZ_B0132 by snippy are reported in Supplementary Tables 5, 6. There is a concentration of putative recombination events toward the end of the genomes (between 999,298 to 999,332 bp), the gene immediately prior to this was identified in UniProt as a variable surface protein (*vsp*).

### 3.3 Characterization of the New Zealand ST21 Outbreak

**FIGURE 6:**
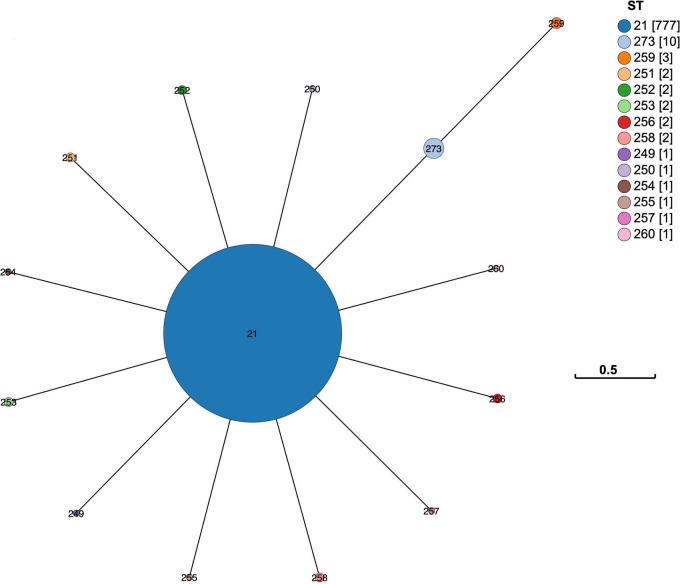
Minimum-spanning tree of the *M. bovis* multilocus sequence types found in the New Zealand ST21 Outbreak. The MST based on the 7-gene MLST profiles for 806 New Zealand genomes shows most are ST21 (*n* = 777) with 29 variants. There are 13 new STs, most of these variants (*n* = 26) are one SNP changes within the MLST loci. Three genomes shared the same one SNP change in the *glt*X loci with ST273 (*n* = 10) and have another one SNP change in the *dna*A loci to become ST259. Overall, this pattern is consistent with a clonal expansion of the ST21 outbreak in New Zealand.

**FIGURE 7:**
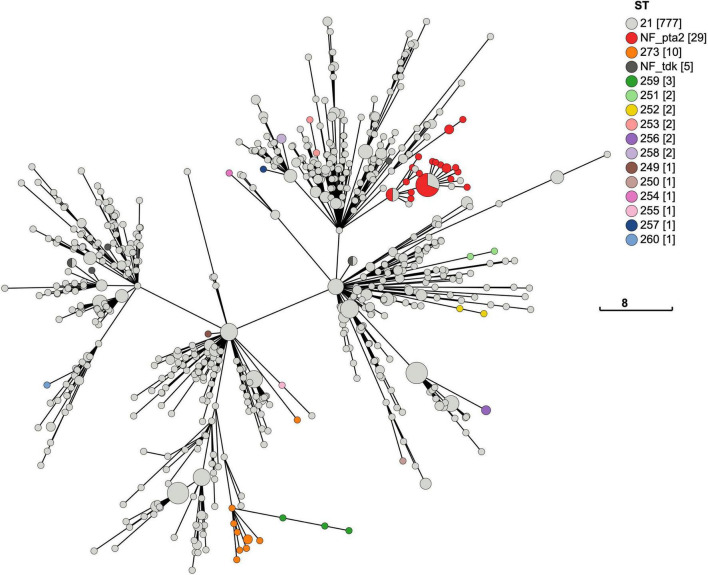
Minimum-spanning tree of the cgMLST for New Zealand *M. bovis*. A MST of the cgMLST for *M. bovis* genomes in New Zealand (*n* = 840). The size of each node represents the number of genomes with the same cgMLST profile made from the 386 shared loci. The 7-loci MLST ST21 dominates (*n* = 777), and the most common variant (*n* = 29) lacks a *in silico* detectable *pta*2 locus (NF_*pta*2). Most genomes with this variant cluster together. Genomes lacking a *tdk* locus (*n* = 5) are called NF_*tdk*. Each new variant with a full MLST profile makes a new ST. The new STs appear to be randomly spread across the MST. But when there is more than one of the same sequence variation or new ST, they tend to cluster together suggesting a lineage. The legend shows how the nodes are colored by the 7-gene MLST scheme developed by Register et al. (2020). The legend has a tally of the total number of genomes for each ST, which are shown in square brackets. The total number of allele differences between each node are represented by the branch length. The scale bar shows the length for a branch with eight allelic differences.

**FIGURE 8:**
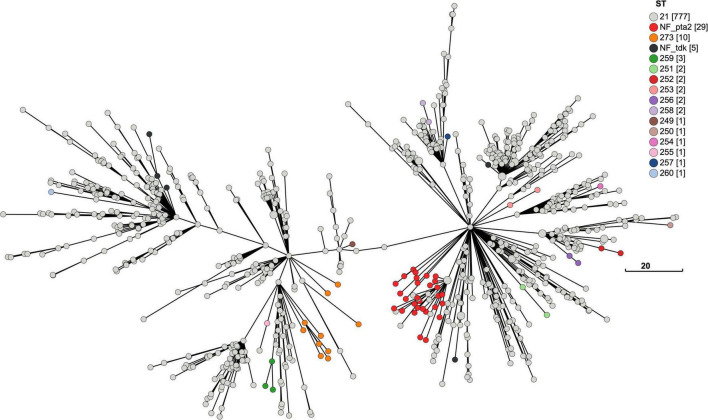
Minimum-spanning tree of the wgMLST for New Zealand *M. bovis.* A MST of the wgMLST for *M. bovis* in New Zealand (*n* = 840). Each node represents a genome with a unique wgMLST profile made from 1340 loci. The wgMLST includes more loci than the cgMLST (1,340 > 386 loci), enabling more variation between the genomes to be shown by including accessory loci that are not present in all the genomes. The occurrence of a new variant sequence within the MLST loci appear to be randomly spread across the MST but when there is more than one of the same new ST variants, they tend to cluster together. The most common variant (*n* = 29) in the 7-gene MLST scheme lacks an *in silico* detectable *pta*2 locus (NF_*pta*2). Most genomes with this variant cluster together. Genomes lacking a *pta*2 locus are called NF_*pta*2 and those lacking a *tdk* locus are called NF_*tdk.* The legend shows the nodes are colored by the 7-gene MLST scheme developed by Register et al. (2020). The legend has a tally of the total number of genomes for each ST, which shown in square brackets. The total number of allele differences between each node are represented by the branch length. The scale bar shows the length for a branch with 20 allelic differences.

The accumulation of changes in the 840 New Zealand *M. bovis* genomes can be seen in the MST of the MLST, cgMLST and wgMLST profiles which are visualized in Figures 6–8, respectively. Each showed evidence of new alleles emerging and radiating out from more central nodes.

During *in silico* genotyping for the MLST, 34 genomes were removed from the original 840 genomes, leaving n = 806, because we were unable to fully identify all seven loci in the MLST profile (Supplementary Table 2). Most (*n* = 777) genomes were ST21, and a small number of genomes (*n* = 29) showed SNP variations to ST21 in the seven MLST loci (Figure 6). There was a total of 13 new STs identified in this analysis of the New Zealand genomes, including the three mentioned above in the ST21 and close variants dataset. Most of the variants (*n* = 26) vary by one SNP from ST21 (ST249, ST250, ST251, SR252, ST253, ST254, ST255, ST256, ST257, ST258, ST260, ST273). Three genomes were ST259 and showed two SNP changes, one in each locus (*dna*A, *glt*X). ST259 shares the same SNP in the *glt*X loci as ST273. This pattern is consistent with the clonal expansion of the ST21 during the outbreak in New Zealand.

The wgMLST for the 840 New Zealand genomes included more loci and therefore it had more opportunity to identify differences and divergence than the cgMLST (1340 vs 386 loci). Panaroo produced a core genome (689 genes) and a pangenome (811 genes). roProfile made a cgMLST (386 loci) and the wgMLST profiles (1,340 loci).

Using the MST format, we reviewed the distribution of mutations within the MLST sequences when each genome of this outbreak is represented by a cgMLST (Figure 7) and wgMLST (Figure 8) profile. While a new mutation may occur anywhere in a genome sequence, genomes with the same change in their MLST sequences tend to cluster together e.g., ST259. The most frequent change detected within the MLST sequence shown in the cgMLST and the wgMLST were 29 genomes without an *in silico* detectable *pta*2 locus. Examination of the Prokka annotated genomes showed that 39 genomes had truncated *pta*2 genes (Supplementary Table 2). In contrast, the SRST2 software, which was used for MLST genotyping, uses a different approach involving mapping the FASTQ files and identified 10/39 as having a full *pta*2 locus. The 29 genomes with this feature formed two close clusters in Figures 7, 8 suggesting this change could form lineages and may be an inherited feature.

## 4 Discussion

Our genomic analysis of the 2017 *Mycoplasma bovis* outbreak in New Zealand shows that the outbreak was caused by genotype ST21. Over a 4 1/2 year period (2017–2022) of the outbreak the genomes reveal evidence of clonal expansion, when examining the MLST, cgMLST and wgMLST MSTs. Our analysis of the ST21 dataset reveals allelic variants in six of the core genes unique to the New Zealand genomes. The six genes that had different sequences in New Zealand genomes are *hae*IIIM: encoding for cytosine-specific methyltransferase; *cys*C: encoding for cysteinyl tRNA synthetase; era: encoding for GTPase Era; *met*K: encoding for S-adenosylmethionine synthase; *par*E: encoding for DNA topoisomerase; and *his*S: encoding for histidine-tRNA ligase. The presence of these alleles only in the New Zealand genomes, could be due to a simple population bottleneck in the original transmission event into New Zealand. Alternatively, it could be the result of selection or genetic drift in the initial stages of the outbreak expansion.

We successfully used the MLST scheme for *M. bovis* from pubMLST and developed by Register et al. (2020), to define and monitor the 2017 ST21 outbreak in New Zealand (Jolley et al., 2018; Register et al., 2020). Although, it is notable that 39 of the 840 New Zealand genomes had a truncated *pta*2 gene when annotated by Prokka, in comparison the SRST2 approach found only 29 did not have a full *pta*2 locus. *pta*2 is a housekeeping gene that is part of the *M. bovis* MLST scheme, so lineages with an undefined *in silico* MLST locus will potentially compromise the utility of the scheme. Previously, with the pubMLST legacy system for *M. bovis*, there were occasionally reported difficulties in detecting the *adh*1 locus (Josi et al., 2018; Tardy et al., 2020). We suggest that further work is required to investigate what affects some of the *M. bovis* housekeeping genes, and how the genome is affected as a whole. One possible cause is ICE or MCT which has been previously cited as a mechanism for the exchange of housekeeping genes between *M. bovis* isolates (Tardy et al., 2015; Garcia-Galan et al., 2022). Many features in the *M. bovis* genome generate variation and plasticity, and our results support the need to use both an MLST scheme and whole genome sequencing when examining an outbreak to investigate in depth any anomalies that arise.

Our results showed a greater difference than might be expected between the size of the core genome (*n* = 689 genes) and pangenome (*n* = 811 genes) produced by Panaroo, when compared to the size of the cgMLST (*n* = 386 loci) and wgMLST profiles (*n* = 1340 loci) produced in roProfile, for the 840 New Zealand genomes. The size of the core genome is much larger than the cgMLST (689 > 386), and the pangenome much smaller than the wgMLST (811 < 1,340). While not the same measurements, it would be expected that the sizes of the core genome and cgMLST should be similar, and the size of the pangenome and wgMLST should be similar. In our smaller dataset for ST21 (*n* = 87 genomes), the Panaroo pangenome size (*n* = 921 genes) is closer to the roProfile wgMLST size (*n* = 1,004 loci). These differences in outcome could be due to the different methods used by Panaroo compared to roProfile to calculate their results, each goes through multiple different steps to calculate these values e.g., roProfile uses Roary to calculate the initial pangenome and Panaroo does not. It is also possible that the effect of these different approaches is not as pronounced when comparing a smaller number of genomes.

The international dataset of genomes when characterized using MLST genotyping or FastBAPS clustering, showed countries with large sample sizes like Canada, Australia, and Denmark, had within each country both closely related and distinctly different genomes. Some of these genomes were closely related to genomes present in other countries. For example, we found ST21 in 10 countries, this global spread has shown ST21 as present in North America, Europe, Asia and now New Zealand in the Southern hemisphere. The genomic evidence shows *M. bovis* is spreading between countries and hemispheres, and just as moving infected cattle between farms can spread this infection, it is possible the international movement of live cattle and/or using imported semen may have a role (Haapala et al., 2018; Yair et al., 2020); although transmission by fomites cannot be ruled out (Piccinini et al., 2015).

Modern agriculture is dependent on the regular movement of livestock. As these movements are a key factor in the dissemination of *M. bovis*, it follows that any national control program must understand these movements to control *M. bovis* and other infectious diseases of cattle. If we cannot rely upon antibiotics or effective vaccines (Lysnyansky and Ayling, 2016), then control and management options become limited to strict hygiene standards and movement restrictions of the infected animals, both with and without clinical signs (Pfützner and Sachse, 1996). We support the view any attempt at a national control plan must address all the sources of transmission. To better understand the containment and control of endemic *M. bovis*, a network analysis of cattle movements should be considered by researchers and such data included when modeling potential control methods (Gates and Woolhouse, 2015). An example of how cattle movement data can be used to model disease risk was described by Hay et al., 2014, who showed the lifetime moving and mixing history of Australian cattle (*n* = 35,131) can affect their risk of being treated for bovine respiratory disease in feedlots (Hay et al., 2014). Exposure to *M. bovis* was shown to be an important risk factor for bovine respiratory disease within the same population (Schibrowski et al., 2018).

Two genomes, BEL_Mb192 and Hun_BM632, showed marked variation to other genomes in the ST21 subset of 87 genomes. This suggests significant genomic variation can accumulate outside of the seven MLST housekeeping loci, in this case, likely due to recombination events. MCT can produce mosaicism in the *M. bovis* genome resulting in different areas of the genome having different phylogenetic histories (Garcia-Galan et al., 2022). If mosaicism is a common event in *M. bovis*, SNP-based WGS analysis as well as allele-based MLST genotyping are needed to understand an outbreak. Future work is required to evaluate how common recombination events are in *M. bovis*, particularly those involving MCT and IS. Research is needed into when, and how, housekeeping gene detection is affected. As well as the extent that genome plasticity (in *M. bovis*) will affect genomic epidemiology, and our understanding of transmission events during an outbreak. This knowledge could inform the sustainable use of antimicrobial treatments, and the composition and deployment of vaccines, should they become available, for *M. bovis* in control and/or eradication programs. Recombination events may or may not impact the efficacy of live attenuated vaccines against *M. bovis* or compromise the accuracy of current diagnostic tools used for its detection. For example, the successful control of *M. gallisepticum* in the poultry sector used several live attenuated vaccines effectively for decades, despite variations in outbreak strains and geographical regions (Bíró et al., 2005; Noormohammadi and Whithear, 2019). Current diagnostic tools for detecting *M. bovis* have proven highly effective across different geographic regions and strain variations, regardless of differences from the original strain used to identify the target gene or protein (Petersen et al., 2018; Wisselink et al., 2019; Salgadu et al., 2025).

The pangenome size for the ST21 dataset of 87 genomes is 921 genes but the NZ dataset of 840 genomes has a smaller pangenome of 811 genes. The ST21 group, although a smaller sample size, is taken from the widely spread global population of ST21. The global population of ST21 has been around for a long period (collected from 2007 to 2020) and accumulating mutations and recombinations, i.e., creating diversity in the pangenome. The large recombination events found in two of the ST21 indicate how these changes are accumulating and adding to the pangenome. The overseas samples of ST21 have the opportunity to recombine with more diverse *M. bovis*, while the NZ *M. bovis* when undergoing intra-species recombination events were limited to within its own clonal expansion. The 840 NZ *M. bovis* are considered to be the result of clonal expansion in a recently introduced bacterium, which probably bottled necked and expanded only for a few years (suggested arrival date 2015–2022). Another contributing factor could have been the nature of the NZ eradication scheme, which was to cull entire infected herds. This meant that while clonal expansion was accumulating changes over a few years, lineages were being pruned as herds were culled.

In conclusion, our study provides insights into the population structure of *M. bovis* at the global level based on 455 genomes from 16 countries. We found two patterns, one of local propagation within a country, and one of international propagation with the same sequence types (ST) shared between geographically distant countries which is consistent with previous findings (Yair et al., 2020). An understanding of the movement patterns of cattle and semen movement nationally and internationally would enable better *M. bovis* disease control and management. There was a clonal expansion of *M. bovis* ST21 in New Zealand. In New Zealand, the cattle population was naïve to *M. bovis*, and an eradication response was undertaken using culling, rather than containment and treatment. This contrasts with the results from France and Denmark showing the emergence of a dominant strain (Tardy et al., 2020; Thézé et al., 2023). In these countries farm husbandry and antibiotics were used to control the infection. Our results suggests that epidemiological evaluations of *M. bovis* requires MLST in combination with WGS analysis to account for the effects of genomic plasticity and mosaicism, which can affect housekeeping loci used in the MLST scheme.

## Data Availability

The sequencing data presented in this study can be found in online repositories at: https://www.ncbi.nlm.nih.gov/bioproject/PRJNA664415, https://www.ncbi.nlm.nih.gov/bioproject/PRJNA 1247756, and https://www.ncbi.nlm.nih.gov/nuccore/CP192245.1/.

## Footnotes

1
https://pubmlst.org/organisms/mycoplasma-bovis

2
https://www.ncbi.nlm.nih.gov/sra/

3
https://github.com/tseemann/snippy

4
https://www.pacb.com/wp-content/uploads/Procedure-Checklist-Using-the-BIO-RAD-CHEF-Mapper-XA-Pulsed-Field-Electrophoresis-System.pdf

5
https://www.geneious.com

## Supplementary material

Supplementary Table 1 The MLST genotype and FastBAPS clusters for 455 genomes from 16 countries and 11 without a known country.

Supplementary Table 2 The MLST genotype and pta truncations for 840 New Zealand genomes.

Supplementary Table 3 The methods used for 163 Non-New Zealand genomes. Including (Sulyok et al., 2014; Haapala et al., 2018; Zhu et al., 2018; Tardy et al., 2020; Triebel et al., 2023).

Supplementary Table 4 The genotype and SNP positions of the 87 ST21 and one SNP variants.

Supplementary Table 5 Putative Recombination events detected in Bel_Mb192. A.gff file shows the fully or partially affected genes at the recombinant event loci when aligned by snippy to NZ_B0132.

Supplementary Table 6 Putative Recombination events detected in Hun_BM632. A.gff file shows the fully or partially affected genes at the recombinant event loci when aligned by snippy to NZ_B0132.

Supplementary Table 7 Putative Virulence- and Adhesion-related genes. The genes are identified as present or absent. The genes are designated with the same name used by Josi et al. (2018).

Supplementary Table 8 The NCBI data for the 444 non-NZ genomes in the international dataset.
